# Supplementary figures and images for: The responses of CO2 emission to nitrogen application and earthworm addition in the soybean cropland
Source: PeerJ. 2024 Mar 26;12:e17176. doi: 10.7717/peerj.17176 (PMC10979750; doi:10.7717/peerj.17176)

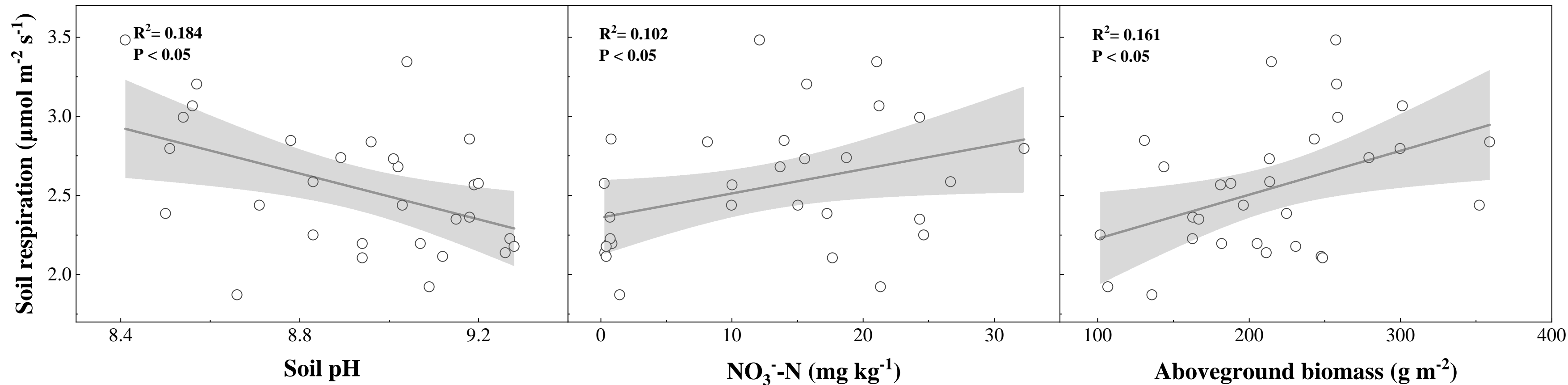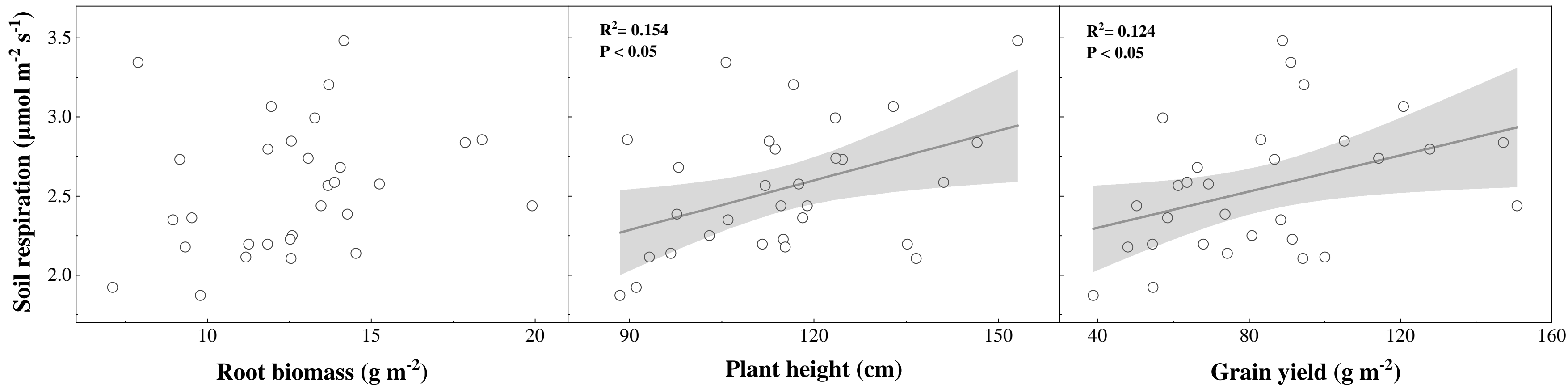

Supplement: Supplemental Information 2 [file peerj-12-17176-s002.pdf]

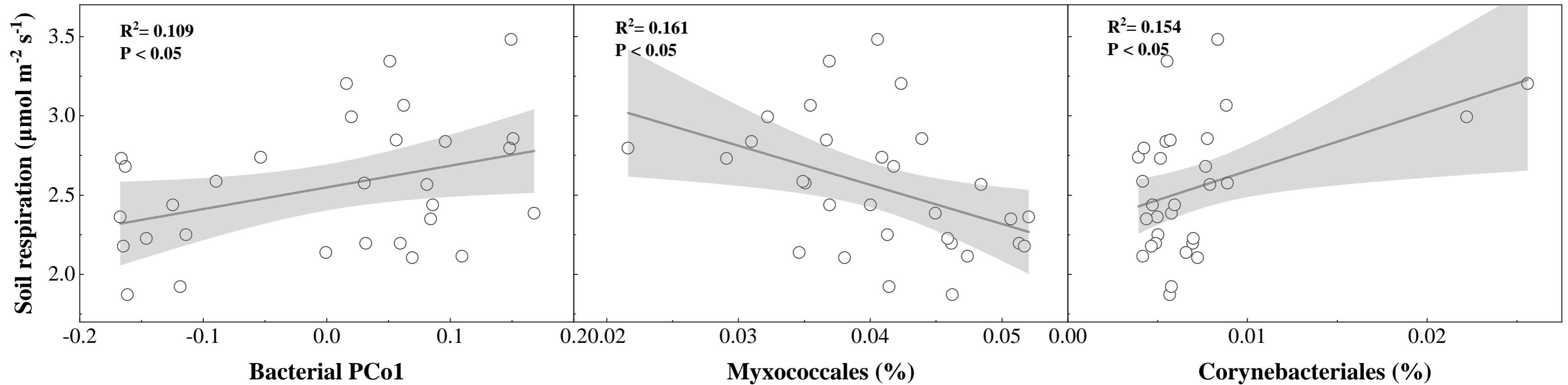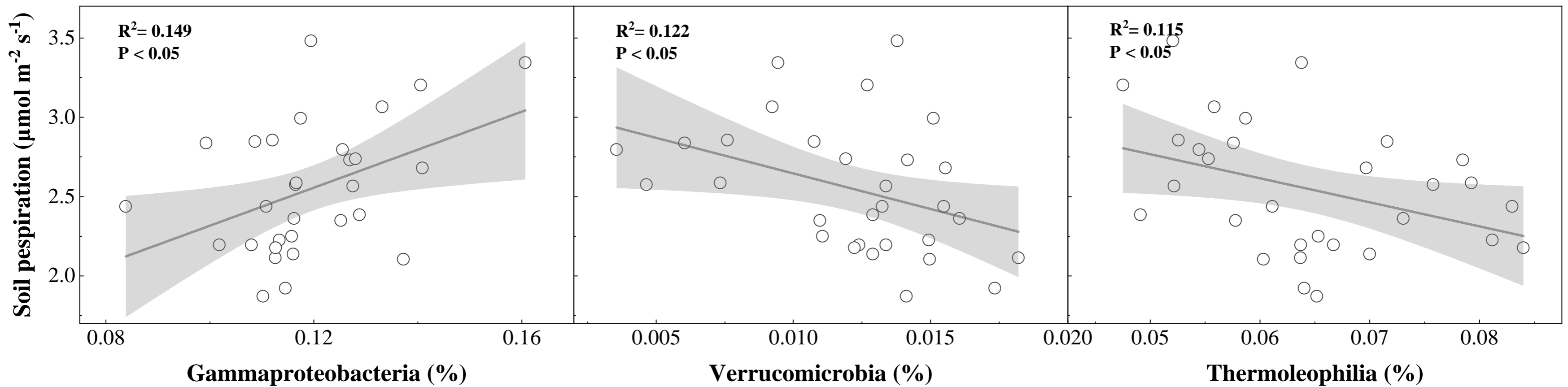

Supplement: Supplemental Information 3 [file peerj-12-17176-s003.pdf]
